# Evaluation of the Feasibility, Reliability, and Repeatability of Welfare Indicators in Free-Roaming Horses: A Pilot Study

**DOI:** 10.3390/ani11071981

**Published:** 2021-07-02

**Authors:** Jessica J. Harley, J. David Stack, Helen Braid, Krista M. McLennan, Christina R. Stanley

**Affiliations:** 1 Animal Behaviour & Welfare Research Group, Department of Biological Sciences, University of Chester, Chester CH1 4BJ, UK; k.mclennan@chester.ac.uk (K.M.M.); christina.stanley@chester.ac.uk (C.R.S.); 2 Faculty of Health and Life Sciences, Institute of Infection, Veterinary & Ecological Sciences, The University of Liverpool, Neston CH64 7TE, UK; J.D.Stack@liverpool.ac.uk (J.D.S.); H.Braid@liverpool.ac.uk (H.B.)

**Keywords:** animal welfare, assessment, equine, extensively managed, feral horses

## Abstract

**Simple Summary:**

Animal welfare assessment is an essential tool for maintaining positive animal wellbeing. Validated welfare assessment protocols have been developed for farm, laboratory, zoo, and companion animals, including horses in managed care. However, wild and free-roaming equines have received relatively little attention, despite populations being found worldwide. In the UK, free-roaming ponies inhabit areas of Exmoor, Dartmoor, and New Forest, England, and Snowdonia National Park in Wales, amongst others. Visitors and local members of the public who encounter free-roaming ponies occasionally raise concerns about their welfare, as they are not provided with additional food, water, or shelter. In this study, we evaluated the feasibility, reliability, and repeatability of welfare indicators that can be applied to a population of free-roaming Carneddau Mountain ponies to address such concerns. Our findings indicate that many of the trialed indicators were successfully repeated and had good levels of inter-assessor reliability. Reliable and repeatable welfare indicators for free-roaming and semi free-roaming ponies will enable population managers and conservation grazing schemes to manage the welfare of free-roaming horses and ponies.

**Abstract:**

Validated assessment protocols have been developed to quantify welfare states for intensively managed sport, pleasure, and working horses. There are few protocols for extensively managed or free-roaming populations. Here, we trialed welfare indicators to ascertain their feasibility, reliability, and repeatability using free-roaming Carneddau Mountain ponies as an example population. The project involved (1) the identification of animal and resource-based measures of welfare from both the literature and discussion with an expert group; (2) testing the feasibility and repeatability of a modified body condition score and mobility score on 34 free-roaming and conservation grazing Carneddau Mountain ponies; and (3) testing a prototype welfare assessment template comprising 12 animal-based and 6 resource-based welfare indicators, with a total of 20 questions, on 35 free-roaming Carneddau Mountain ponies to quantify inter-assessor reliability and repeatability. This pilot study revealed that many of the indicators were successfully repeatable and had good levels of inter-assessor reliability. Some of the indicators could not be verified for reliability due to low/absent occurrence. The results indicated that many animal and resource-based indicators commonly used in intensively managed equine settings could be measured in-range with minor modifications. This study is an initial step toward validating a much-needed tool for the welfare assessment of free-roaming and conservation grazing ponies.

## 1. Introduction

Knowledge of the welfare of animals under human care is integral to their successful management; equally important is an understanding of the welfare of free-living animals to guide how we interact with wildlife and their habitats [1]. To gather knowledge to improve animals’ welfare, a validated, reliable, and repeatable method of assessment is required [2,3]. Recently, welfare assessment has moved from resource-based or simple indicators of environmental parameters to include indicators that monitor the behavioral responses and physiological conditions of individual animals over time [4]. Animal-based indicators are particularly relevant in the welfare assessment of wild or free-ranging animals. Indicators related only to environmental parameters do not allow for the assessment of the behavioral or physical responses to the prescribed condition and are not representative of the animal’s welfare state [4]. An animal’s ability to adjust to both predictable and unpredictable change in its environment is vital to maintaining welfare [5]. For example, seasonal changes in forage and grass availability for grazing animals may elicit periods of fasting (hunger) or seasonal weight gain (fat reserves) to cope with available resources [4]. While periods of hunger may be considered a welfare issue, this may not be a factor for the animal itself if its adaptive capacity (physical and mental abilities) has not been exceeded [4]. Using a multifactorial approach including animal-based indicators (AB) (physical/physiological outcomes) and resource-based indicators (RB) (what is available in the environment) for assessment enables the evaluator to quantify levels of individual welfare [6]. There are a range of welfare audit protocols that have recently been developed to determine welfare by evaluating RB and AB indicators for farm [7,8], companion [9], laboratory [10], and zoo animals [11,12,13,14]. In contrast, there are few protocols for extensively managed animals [3,15,16] or, indeed, free-living wild populations [1].

Populations of free-living and free-roaming horses are found throughout the world. Significant numbers occur in Australia, with an estimate of over 300,000 free-roaming brumbies [17]. In the US, smaller populations of free-roaming horses occur. There are just over 79,000 mustangs and 15,546 burros managed by the United States Department of the Interior’s Bureau of Land Management (BLM), inhabiting approximately 31.5 million acres of land across 10 states in the Western United States [18]. In the UK, free-roaming ponies inhabit areas of Exmoor, Dartmoor, New Forest in England, and the Carneddau mountains in Wales, amongst others.

Public concern regarding the welfare of free-roaming equids has become more prevalent in recent years. Whilst increased public awareness and demand for the improvement of equine welfare are evident across various equine disciplines, e.g., sport, working horses, racehorses, and those kept for pleasure [19,20], there is also concern regarding the welfare of feral populations. Equine stakeholders participating in a study carried out by Horseman et al. [20] identified overbreeding, a lack of food in winter, and gatherings (rounding-up for health checks) as areas of welfare concern for free-roaming ponies specifically. The public’s concern for the welfare of mustangs in the US has been particularly well-documented, with many groups urging the Bureau of Land Management to cease all gatherings, removals and contraceptive strategies [21]. Visitors and local members of the public who encounter free-ranging ponies in the Carneddau mountains also occasionally raise concerns about their welfare because they are not provided with additional food, water, or shelter (Carneddau Pony Society, personal communication, 3 November 2019). There is therefore a need for objective indicators of welfare in these populations at both the individual and group levels. Despite several validated equine welfare assessment protocols in existence for sport, pleasure, and working horses, e.g., [22,23,24,25,26], there is currently only one audit available for free-roaming horses. This describes a 10-step protocol using the Five Domains model that can be used to form a template for welfare assessment in free-living terrestrial species and uses Australia’s brumby horses as an example [1]. Here, we therefore trialed specific welfare indicators to ascertain their feasibility, reliability, and repeatability for the welfare assessment of free-living horses using free-roaming Carneddau Mountain ponies as an example population.

## 2. Materials and Methods

### 2.1. Ethics 

This study was granted ethical approval by the University of Chester’s Faculty of Medicine and Life Sciences Research Ethics Committee on 6 February 2020, reference number 1609/19/JH/BS. The Carneddau Mountain Pony Society provided written permission to access the study population on 3 November 2019.

### 2.2. Study Population

The Carneddau Mountain ponies are classified as semi-wild (surviving and breeding without human intervention) and believed to be free-roaming since the Bronze Age [27]. Approximately 220 free-roaming ponies comprising mares, their offspring, and 12–15 dominant stallions inhabit about 5377 hectares of habitat [28]. Genetic studies have revealed that the Carneddau Mountain ponies are genetically distinct and isolated from other Welsh pony populations, and they could therefore represent a valuable future genetic resource [29]. The ponies graze on common land; families whose homesteads border the Carneddau mountains are each entitled to grazing rights under the Common Rules 1966 [30], and members of nine families with rights to graze ponies on the Carneddau mountains are represented in the Carneddau Mountain Pony Society, a non-profit organization responsible for managing the Carneddau ponies. This population is therefore semi-feral; it is not heavily managed, yet each pony is privately owned.

### 2.3. Choosing the Welfare Indicators 

A systematic review of existing equine assessment literature identified several AB and RB indicators of welfare feasible for free-roaming ponies [1,19,23,24,25,31,32,33,34,35]. Management indicators were excluded from consideration because the subjects are free-roaming, and management intervention in-range is minimal. Each identified AB and RB indicator was reviewed and discussed by the lead investigator with a small team of experts in the UK. Experts included a first opinion equine veterinarian (BVetMed and MRCVS); a specialist equine surgeon (MVB, MSc, DipECVS, and MRCVS); a behavior, equine science, and farm animal welfare scientist (PhD); a welfare scientist with wild animal experience (PhD); a conservation grazing scheme manager with extensive knowledge of the Carneddau landscape and ponies; and a behavior and welfare scientist with wild animal and equine experience and a working knowledge of the Carneddau ponies (PhD). Indicators were discussed based on criteria of significance to free-roaming equine welfare, feasibility, and practical application of the indicator in-range for non-specialist’s stock persons and grazing scheme managers, as well as their validity in terms of evidence from the published literature [19,31,34,35,36].

In total, 12 AB and six RB welfare indicators were chosen for further evaluation. Most welfare indicators were either categorized with a zero or one to represent a negative state of different degrees of severity, followed by two = neutral, and sometimes three = positive. This type of multi-level numerical scoring system has been used in several of the published welfare assessment templates, e.g., the WQ® system [8] and the Standardised Equine Based Welfare Assessment (SEBWAT) [26]; however, in WQ^®^, zero = positive, 1 = neutral, and 2 = negative, and in SEBWAT, they use a mixed approach for scoring criteria. In this study, based on the intended end-users, we determined that it was more intuitive to have a higher number equate to a positive category. If a score of zero was applied, a significant risk of welfare compromise was indicated and escalation was required (i.e., reporting the pony to the population managers). A few indicators were categorized using letter scores alone. Reproductive status was classified as lactating, not lactating, or not applicable (e.g., sub-adult female and stallion). Hoof shape was classified as (A) for long overgrown hooves, (B) for hoof cracks (vertical/horizontal), and (N) for normal. Fecal consistency was classified using a mixture of numerical and letter categories; (N) was assigned for normal feces, and feces that were not well-formed, cow-dung-like, or mostly comprised large fibers received an (A) for abnormal. However, feces that had a water-like consistency (diarrhea) received a category of zero. This mixed approach was used for feces due to the rapid progression of the disease state (dehydration, electrolyte derangement, and endotoxemia) that can accompany acute diarrhea resulting from typhlocolitis in horses. Acute diarrhea resulting from typhlocolitis is a major clinical sign observed in horses with salmonellosis, intestinal clostridiosis, cyathostomiasis, and (to a lesser extent) strongylosis [37].

Though some indicators could only be assigned a score of 1 or above, others had the option of a zero score because these were deemed to indicate a more severe level of welfare compromise. Those that include an option of zero included BCS, mobility, ocular discharge/swelling, and wounds/swelling. If a pony received a zero in any of these categories, the “second level” application of the Horse Grimace Scale (HGS) was indicated using the HGS mobile application, in addition to reporting the issue to the Carneddau Pony Society. This process of classification ensured that the correct weighting was assigned to a welfare indicator that posed an immediate danger to the health and welfare of the pony, e.g., this assured that alopecia of mane/tail was not weighted at the same level as an open wound > 7 cm involving deep tissue and muscle. The HGS also had a zero score option. All remaining indicators are outlined in Table 1.

### 2.4. Feasibility of Assessing Welfare Indicators In-Range

The majority of welfare indicators were derived from literature focusing on the welfare assessment of intensively managed horses (stabled); therefore, it was essential to determine whether the selected indicators could be carried out in-range and whether the minor modifications that would be required for in-range setting were appropriate. All 12 AB and six RB indicators were explored and tested in-range at conservation grazing sites and in the Carneddau Mountain on free-roaming ponies prior to the main testing phase to confirm their feasibility. This enabled us to develop a decision tree-based question for each of these welfare indicators. The pre-testing also enabled the identification of auxiliary equipment necessary to facilitate observations of ponies, e.g., binoculars. Finally, any significantly modified indicator, e.g., body condition score (BCS) or mobility, was individually tested for observer reliability during a pre-trial of indicators.

**Figure 1:**
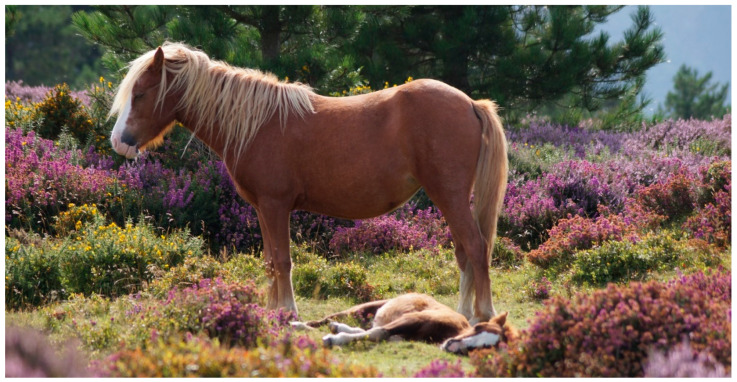
Carneddau pony mare with foal at foot. The image illustrates the difficulty of accessing hoof shape. Patches of grass are surrounded by heather (*Calluna vulgaris*) and gorse (*Ulex europaeus*).

In an early stage of the study, it was determined that forage availability, quality and accessibility were difficult to evaluate. For an assessor to achieve an objective appraisal of resource provision, it would be time-consuming and require specialist knowledge of the species of plants in-range and those which are consumed by the ponies, so it was not practical for a rapid welfare assessment in a free-roaming habitat. Therefore, it was not included in the list of RB indicators for nutrition, and the focus was placed on BCS as the primary AB indicator for nutrition. Hoof shape was feasible in-range, but it was also a time-consuming indicator, as the ability to quantify this required extended focal observations; therefore, this was only included as a second level requirement if the pony’s mobility was impaired (Figure 1). Fecal samples were opportunistically observed, and despite not having a time limit for observations, waiting for a pony to defecate could potentially add considerable time to an individual pony observation. For those ponies that did defecate, feces were easy to score and collect without moving into a pony’s flight zone. Pictures were taken of defecating ponies along with landmarks (e.g., protruding rocks and vegetation) in proximity to the fresh feces. Photos were then used to locate feces and assess fecal consistency.

Ponies were photographed during initial observations, and their GPS locations were recorded to enable the identification of ponies on subsequent visits. All ponies were easily located on each visit, with some nursing mares using close to the same grazing area during both visits. All ponies were approachable with varying flight zones, the majority of which were between three and nine meters. The Carneddau Mountains are a tourist destination attracting walkers, fell runners, and mountain bikers, and the ponies are exposed to regular human–animal disturbance. Ponies were never given less than three meters of space on approach; however, during observations, several ponies came within a meter or less of observers, as foals were curious, and adults would approach while grazing. Binoculars were used to assess several the indicators regardless of distance and were required for the assessment of wounds, nasal discharge, ocular discharge, skin condition, and hoof shape. Both eyes and nostrils were not always visible on first approach as due to the position of the pony. However, the observer could easily move around the pony to achieve the best vantage point. The upland conditions provide open spaces, so the pony moving out of sight of the observer was not a factor. Binoculars were also used to identify bands from a distance when locating individual ponies. The most important aspect of the assessment of the welfare indicators in-range was allowing for ample time to assess. It was often necessary to wait until a pony moved to an area with open patches of grass or to one of the paths or roads (which they frequently accessed) to observe lower limbs.

### 2.5. Modification and Pre-Testing of Welfare Indicators for In-Range Assessment

#### 2.5.1. Lameness and Mobility

Lameness and compromised gait scoring are animal-based indicators found in numerous equine welfare assessments for sport and working horses and donkeys, e.g., [22,23,25,26,33]. A typical equine lameness examination includes horses observed in the walk and trot on flat even ground, in a straight line, and circling on a hard surface [38]. Like many environments in which free-roaming ponies inhabit, the Carneddau Mountain terrain is steep, uneven, and features tall vegetation and varying substrates. Free-roaming horses are also not frequently handled, which makes an in-hand lameness evaluation prohibitive. Thus, a standardized lameness grading protocol would not be feasible in-range, meaning a scoring system focusing on the pony’s mobility rather than identifying a specific type of lameness was required.

**Figure 2:**
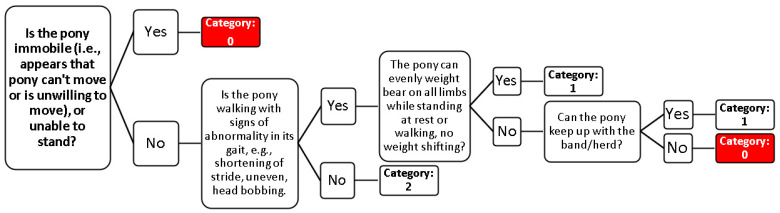
Mobility decision tree from the Carneddau Mountain pony welfare indicators template.

The mobility scoring system used here was modified from the Agriculture and Horticulture Development Board, UK (AHDB) Dairy Mobility Score system [39], which indicates a cow’s ability to move comfortably in a walk during normal, unaided locomotion for 6–10 strides, observed both from the side and behind the animal. The system scores each cow on a 3-point scale; cows with good mobility (no impairment) are scored as zero, imperfect mobility (steps unevenly) is scored as 1, and cows with impaired mobility (cannot keep up with herd and limb lameness is visible) are scored as two. Similarly, we used a 3-point scale from 0 to 2, with a score of two indicating no signs of abnormality in mobility, 1 indicating walking with abnormality in gait and not even in rhythm (weight-bearing), and zero signaling severely impaired mobility (pony unable or unwilling to move forward, unable to stay with herd). Individual ponies were observed from a suitable distance (respecting the ponies’ flight zones) that was not less than three meters. In practice, this was usually less than nine meters (binoculars could also be used as required), and for the majority of ponies, this was just over three meters. The ponies were observed resting and then in a natural walk, with the assessor viewing from the side and rear of the pony. Assessors did not push the pony forward by entering the flight zone; instead, they observed until the pony walked freely and naturally and scored based on findings. There was no time limit for the observation. The observer then used the decision tree (Figure 2) to score the pony. A score of zero required immediate action, as previously indicated. If the pony was not moving for a non-musculoskeletal issue such as colic or being stuck (e.g., in a ditch), then the mobility was not scored and immediate action was taken.

#### 2.5.2. Body Condition Score (BCS)

Equine body condition is commonly assessed using either a 5-point [40] or 9-Point [41] scale. Scoring is commonly performed both visually and using palpation to evaluate body fat and muscle covering specific areas of the body, including the neck, shoulder, ribs, abdomen, and rump. A BCS is a valid, reliable and repeatable measure to assess on-farm nutrition [19]. However, palpation on free-roaming ponies is often not feasible. Body condition scoring is sometimes carried out by comparing the equine in question with pictures [42,43] or observations without palpation [44]. Data collection commenced with an attempt to validate BCS by comparing assessor scores of the same pony with and without palpation. Two groups of conservation grazing ponies were selected *n* = 22; both facilities had race and crush systems in place for safe handling and had previously handled the ponies. However, during our initial assessment, it became evident that the ponies had not been sufficiently handled to enable safe palpation (for pony/assessor), nor was the crush system adequate for the ponies at one facility. The decision was made to suspend the validation of BCS scoring without palpation and to focus on the inter-reliability of assessors using a modified (no palpation) approach. Before the commencement of the formal study, we conducted a pre-trial of BCS without palpation by two assessors (a primary investigator and an equine veterinarian) on thirty-four free-roaming and conservation grazing ponies between February and March 2020. The observer approached the pony slowly and quietly without entering the pony’s flight zone (if the pony turned or moved away, the observer stopped immediately, as this indicated they had entered its flight zone). Observations were typically about three meters from the pony, although a handful of ponies had slightly larger flight zones (both observers viewed from the same point). The observer began with an initial visual inspection from the side of the pony to examine muscle and fat cover of the ribs, neck, shoulder, back, abdomen, and pelvis. The observer then slowly and quietly stood behind the horse and evaluated fat deposits around the tail bone while observing the shape of the croup (the point of the buttock). Finally, the observer assessed the visibility of the spine and hip bone. The observer then referred to the body condition score template [41] using a scale from one (extremely emaciated) to nine (extremely fat) to score the pony’s body condition. This score was then categorized as a 3-point score (from 0 to 2). Ponies scoring a zero in the modified BCS equated to a Henneke score of 1–2 (emaciated/poor) or 8–9 (fat/extremely fat); as both emaciation and obesity have welfare implications, a score of one was equal to thin–moderately thin (Henneke score: 3–4) and a score of 2 equated to moderate (Henneke score: 5–7) [41]. Pictures for the later identification of each pony were taken during the BCS scoring.

#### 2.5.3. Horse Grimace Scale (HGS)

Pain in animals is an unpleasant sensory and emotional experience, and it is a significant welfare concern [45,46]. Methods of the assessment of pain are essential for animal management. Facial expressions as an indicator of pain have been defined for several species including sheep [47], rabbits [48], rodents [49], and horses [31]. The HGS was validated for use in horses undergoing routine castration and has been used in the AWIN Welfare Assessment for Horses [33]. The HGS was developed into an application that is available for most mobile phone platforms (e.g., IOS and Android) and includes in-built training for users. In this welfare assessment trial, HGS was indicated as a second level assessment for ponies scoring a zero for the following AB indicators: mobility, ocular injury/discharge, wounds, fecal consistency, and BCS. If a zero was scored, the HGS mobile application was accessed and the assessor was asked to answer all questions in the HGS 13-point scale, which resulted in a possible HGS score from 0 to 12. A score of zero equated to no facial indicators of pain present and a score of 12 indicated that all equine facial indicators of pain were obviously present to the assessor. The assessor was then asked to classify the HGS score according to a 3-point scale as follows: an HGS score of 0–3 received a score of 2, an HGS score of 4–7 received a 1, and an HGS score of 8–12 resulted in a score of zero.

### 2.6. Testing the Welfare Indicators for Reliability and Repeatablility

The final template comprised 17 questions that received a numerical score between 0 and 3, two categorized questions where the observer applied a letter score (hoof condition and reproductive status), and one that used a mixed method (fecal consistency) for a total of 20 questions. In July and August 2020, the prototype assessment template was trialed on 35 free-roaming Carneddau mountain ponies across two grazing areas on the Carneddau mountains: Abergwyngregyn/Llanfairfechan (53.2286705, −3.9842733) and Conwy (53.272243, −3.868534) common areas. Individual ponies were concurrently evaluated by two people (a primary investigator and either an equine veterinarian or a behavior and welfare scientist with equine experience) to test the reliability of each indicator. Of the thirty-five assessed ponies, twenty-six were adults, six were sub-adults, and three were foals, with a sex ratio of twelve males to twenty-three females, representing six bands, a bachelor group, and a mare and foal pair. The female bias was a result of the composition of the study population, as this comprises approximately fifteen stallions and only a few bachelor bands. All assessments of welfare indicators were conducted between 08:30 and 18:00 h and were not carried out during heavy rain to avoid conditions that would affect visibility and assessor safety on the mountain, as permissible within the mountain safety advice for Snowdonia National Park.

In addition, the welfare indicators were tested and repeated on twenty ponies by the primary investigator within 7–17 days of the original assessment to test for the repeatability of each indicator. Ponies were photographed for identification at the commencement of the evaluation to ensure that ponies could be recognized (by individual markings) on future visits to test for repeatability. All repeat assessments were conducted on ponies in the Abergwyngregyn and Llanfairfechan common area.

### 2.7. Statistical Analysis 

All statistical analyses were carried out using the R 4.0.2 platform (Vienna, Austria) [50] and MedCalc for Windows, version 19.2.3. (MedCalc Software, Ostend, Belgium) ([51] Reliability and agreement considerations were selected as advised in the literature for assessing inter and intra-rater variability [52,53,54,55].

#### 2.7.1. Preliminary Testing of Modified BCS and Mobility Descriptors for Inter-Assessor Reliability

An interrater reliability analysis using the Cohen’s kappa (k) and weighted kappa (kw) test statistic was performed to determine consistency among assessors scoring each pony in the preliminary testing phase using the modified Henneke BCS system and the mobility score system. The kappa statistic compares the observed agreement between two assessors on an ordinal scale using a chance-adjusted indicator, and it considers the matches on the main diagonal [52]. The weighted kappa is modified such that it considers off-diagonal differences between observers and considers the degree of disagreement between observers rather than treated as equal, and it is preferable for ordinal data. Linear weighting based on agreement was used because the questions were all based on a 3-point scale. Linear weighting places the same importance on the difference between the first and second category as the difference between the second and third category [53]. Values were interpreted according to the work of Altman [54] with values of 0.81–1.00 considered very good, 0.61–0.80 considered good, 0.41–0.60 considered moderate, 0.21–0.40 considered fair, and <20 considered poor agreement. Kappa estimates and their 95% confidence intervals were calculated using the “psych” package in R [56].

#### 2.7.2. Final Testing of All Welfare Indicators (Prototype Template) for Inter-Assessor and Test/Retest Reliability

Analysis for inter-assessor agreement between the primary assessor and one of three additional assessors using the prototype welfare assessment (*n* = 35) was conducted using the linear weighted Cohen’s kappa and the percentage of agreement. The test/retest analysis of the prototype welfare assessment (*n* = 20) by the primary assessor was performed using Cohen’s unweighted kappa test statistic and the percentage of agreement. Values were interpreted according to the work of Altman [54] with values of 0.81–1.00 considered very good, 0.61–0.80 considered good, 0.41–0.60 considered moderate, 0.21–0.40 considered fair, and <20 considered poor agreement. Kappa estimates and their 95% confidence intervals were calculated using the “psych” package in R [56].

## 3. Results

### 3.1. Preliminary Testing of Individual Indicators: BCS and Mobility

There was a good degree of reliability between the two observers for BCS of the 34 ponies tested in the preliminary phase using the modified Henneke score. Cohen’s weighted kappa score was 0.78 (95% CI: 0.78–0.78; (Table 2). The reliability of the mobility scoring using a 3-point scale was very good, with 100% agreement between the two observers in their scoring of the 34 ponies and a κ_w_ of 1 (Table 2).

### 3.2. Inter-Assessor Reliability

In this study, several of the welfare indicators in the audit were not witnessed (alopecia of mane or tail, nasal discharge, and coughing). They were therefore not included in the results because their reliability could not be determined. The HGS [31] was only infrequently indicated as the prescribed criterion for conducting the HGS required the observed pony to score a zero in one or more of the following categories: mobility, ocular injury/discharge, wounds, fecal consistency, and BCS. Therefore, assumptions about reliability could not be made. Hoof shape/condition assessment was only indicated in two instances where mobility was impaired. The agreement in these instances was 100% between assessors, but not all specific categories were observed (A = overgrown and B = cracked/chipped) and therefore could not be tested for reliability. Data on fecal consistency were opportunistically collected, and only 12 of the 35 ponies defecated. Assessors reached 100% agreement in terms of fecal consistency assessment, but all ponies had normal feces; other categories (0 = watery, and 1 = abnormal) were not observed, and thus assumptions about reliability across all categories could not be confirmed. Water quality scored three (fresh spring forming a stream, pond, or lake) by all assessors for *n* = 35 ponies. As the categories 1 = no water detected and 2 = stagnant pool/ puddle were not identified, this indicator could not be further tested for reliability. Ocular discharge and/or swelling were only infrequently encountered, and while all assessors had 100% agreement, category 1 = discharge with an open eye was not witnessed. Environment ease of movement (people/bikes/dogs) had 100% agreement between all assessors; however, there were no scores in category 1—high footfall. This was also true for wounds and swelling, where no ponies received a score of 0 = open wound involving deeper tissue/muscle (acute).

The indicators that could be fully evaluated showed mixed reliability across the assessors (Table 3). Assessor 1 (primary investigator) and Assessor 2 (equine surgeon), with *n* = 18 assessments, had a very good agreement between five of the seven remaining indicators (BCS, ease of movement, social contact, and human approach), moderate agreement for comfort around resting, and poor agreement for thermal comfort. Assessors 1 and 3 (scientist), with *n* = 11 assessments, achieved good to very good reliability for social contact and human approach. However, for ease of movement, comfort around resting, and thermal comfort, both assessors assigned scores in only one category, meaning that although they had 100% agreement, assumptions about reliability across all categories could not be made. Finally, Assessors 1 and 4 (scientist), with *n* = 6 assessments, had good to very good agreement (BCS, social contact, and human approach), whilst thermal environment and comfort around resting had kappa scores of 0, indicating no agreement better than chance. Indicators that achieved 100% reliability between assessors were not further investigated; details are provided in Table 4.

### 3.3. Intra-Assessor Reliability (Test/Retest)

As with the inter-assessor trial, during the test/retest phase, some of the welfare indicators were not observed (alopecia of mane or tail, nasal discharge, and coughing). The HGS and hoof condition were not warranted because no ponies scored a zero in any of the indicators that triggered the need to carry out the HGS. Additionally, there were no mobility scores of 0 = immobile or 1 = minor impairment during the test/retest phase. As with the inter-assessor reliability assessment, during the test/retest phase, all ponies scored a three for water quality and availability; therefore, all categories could not be tested for reliability but repeatability was confirmed. For the remaining indicators, the reliability of the repeated observations by the primary investigator of the twenty ponies had mixed results. BCS, reproductive status, and skin condition all had a kappa estimate of 1.0 (very good agreement). Wounds and swelling had fair to good agreement, as did the ease of movement (dogs/bikes/people). Social contact had a kappa score of 0; however, the agreement was 95%, with 19 of the 20 ponies receiving the same score in the initial and repeated welfare assessments. Similarly, comfort around resting had a percentage agreement of 90% and no agreement for kappa, with 18 of 20 ponies receiving the same score in the test/retest phases. All other indicators attained moderate reliability (Table 5).

## 4. Discussion

**Figure 3:**
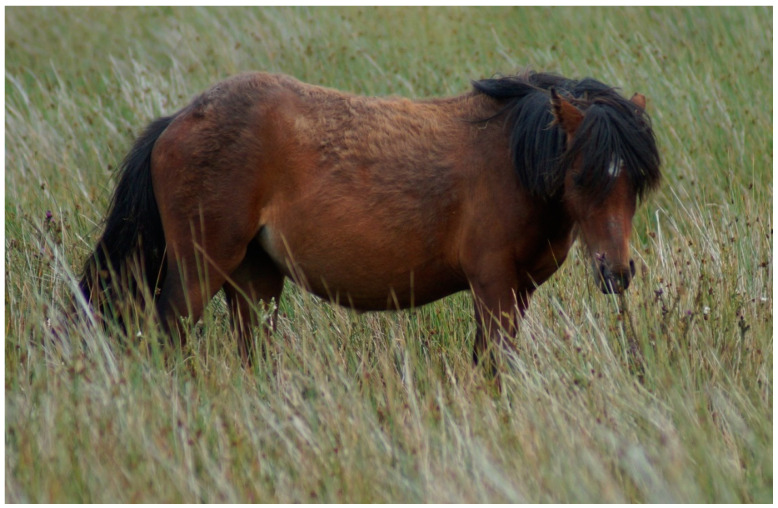
Coat/skin condition achieved very good reliability between observers. Picture is a sub-adult with poor coat quality taken August 2020. This particular band had to be monitored for an extended period of time due to significant time grazing in marshy area, with tall grasses that prohibited view of limbs for wounds/swelling, mobility, and hoof quality.

This project involved testing AB and RB welfare indicators to determine their feasibility, reliability, and repeatability. This was carried out to address the need for reliable in-range welfare indicators for free-roaming and extensively managed horses, which, to date, has not been readily available. The results of this preliminary study indicate that many of the indicators trialed here were successfully repeated over two assessments and had good inter-assessor reliability when trialed by the primary investigator, equine veterinarian, and two animal behavior and welfare scientists. Many of the indicators reached the predefined thresholds for reliability (good to very good) between assessors in numerous categories (human approach, BCS, ease of movement, and social contact), and several indicators, such as skin/coat condition, had 100% agreement between assessors (Figure 3).

The resource-based indicators for ease of movement (hazards), comfort around resting, and thermal environment had variable reliability. Each of these questions asked the assessor to view a 500 m circular radius to identify artificial hazards, suitable area for resting, level of anthropogenic disturbance, and access to shelter or shade (microclimates), as well as to use a decision tree-style question to determine the most appropriate answer. Unlike most of the welfare indicator questions, no images were included, which may have allowed for a greater level of subjective interpretation. A stone wall may act as a wind protector and provide a shaded area but is an atypical ‘shelter’ and may be discounted if the observer is only considering more traditional means of shade, e.g., trees. Additionally, without explicitly providing the number of people that quantifies ‘high public footfall,’ the questions are open to subjective interpretation. The addition of visual cues for these questions and quantitative values may be beneficial to improve agreement among assessors and can be easily rectified in future trialing of the assessment. As recreation can negatively and directly affect wildlife, resulting in altered behavior and temporal avoidance of paths and trails [57], this is an important indicator for free-roaming horses that inhabit tourist destinations. Further refinement is required to ensure reliability among observers.

Several indicators were only infrequently assessed (hoof shape/quality, eye discharge, and second level HGS) or not witnessed (alopecia mane or tail, nasal discharge, and coughing), or scores were not obtained across all categories (water quality, fecal consistency, and wounds and swelling). The reliability of these indicators was therefore difficult to fully ascertain in this study. However, we were able to view each pony sufficiently to determine their presence or absence. Occurrences of some of the health indicators in free-roaming populations may also have low prevalence. For example, coughing is a clinical sign typically observed in domestic horses and ponies with equine asthma or pleuropneumonia [37]. The former is often attributed to poor ventilation and increased environmental allergens found in intensively managed environments. The absence of coughing observed in this study reflects the low prevalence of respiratory disease in this population and may be attributed to the open-air environment and low levels of aerosolized allergens. Eye abnormalities were only infrequently observed in the Carneddau population. In contrast, this was the most frequent physical health indicator observed among 75 long-lined tethered horses and in 28% of a population of 112 free-roaming domestic horses kept on public land in South Wales [32]. Where eye injuries do occur, they are painful; corneal ulcerations are common in horses, and bacterial and fungal keratitis may present with initially mild clinical signs, but serious ocular complications may occur without action [57]. The identification of these health indicators, or at least the confirmation of the absence of injuries, is therefore important for the management of free-roaming and conservation grazing ponies. Therefore, one of the important outcomes of this preliminary trial was that assessors were able to view both eyes of each of the observed ponies using binoculars with distances of three-to-nine meters. Assessors at this distance identified both normal eyes and ponies with discharge with the partial or complete closure of the eye with 100% agreement. Another indicator with low variance was water quality. All ponies had access to numerous clean water points comprised spring-fed streams and ponds. Precipitation totals during July and August 2020 were 110.1 and 163.4 mm, respectively [58]. However, the lowest rainfall on record since 1873 was recorded in May 2020, with 12.9 mm falling North West England and Wales [59,60]. Assessments in May could therefore have yielded a different result. Indeed, free-roaming environments are characterized by seasonal variation, and indicators that can identify welfare change across periods impacted by climatic conditions, food availability, and seasonal biological factors (e.g., breeding) are essential. In conclusion, the lack of the incidence of the specific categories in some welfare indicators does not necessarily diminish their value as part of the assessment but rather indicates that some conditions may have low prevalence in this population. Further trialing across different horse populations and seasons in later stages of this project could enable validation in all categories. While testing for reliability was difficult due to homogeneity in some scores, the same difficulties have arisen in equine assessment trials in domestic horses, e.g., [25]. Conversely, the lack of negative scores for indicators of systemic illness or injury may be a positive sign that the population was mostly experiencing higher welfare during the assessment period.

Statistical parameters indicated moderate to very good reliability across the majority of individual indicators in the test/retest phase. A limitation of the Cohen’s weighted kappa coefficient statistical test for reliability is related to the prevalence of the condition under consideration and that low prevalence (skewing of data) affects kappa estimates [61,62,63]. Comfort around resting and social contact had no agreement when using the kappa calculation, but both had high percentages of agreement (90% and 95%, respectively). Skewed scoring, where there is only one score or a low occurrence of other scores, appears to be commonplace in welfare assessment [3,25,64]. Though the percentage of agreement does not correct for any agreement that could occur by chance, all assessors were trained and experienced, therefore reducing the likelihood that a rater would guess and a result would be overestimated. Percentage agreement has been used in other welfare assessments in conjunction with additional reliability tests, as presented in this study [3,25]. This is a reason why the interpretation of kappa values must be carefully considered and why presenting kappa estimates along with the percentage of agreement for the context of how the assessors scored each indicator is useful [55]. However, we could also explain the lack of agreement with comfort around resting and social contact in a test/retest setting because of changes in the dynamic of the group, not as a failure of the indicator to be reliably repeated. For example, in an initial assessment of one stallion, he was observed away from his band overmarking feces, whilst in the follow-up assessment, he had re-joined his band. In this case, it was a change in condition rather than a lack of reliability. Though Carneddau ponies maintain a small, consistent home range of around 1.5 km^2^ [30,65], individuals are unlikely to be in exactly the same area for both assessments.

Following an initial trial carried out on 34 ponies, the resulting modified BCS without the palpation criterion showed a very good level of reliability between assessors, with two assessors and the lead investigator achieving 100% agreement. Within the context of this assessment, BCS is particularly relevant due to the absence of other rapid resource-based indicators to evaluate nutritional state. Thus, the BCS indicator is an integral aspect of the effectiveness of this assessment. However, further testing across the late autumn and winter seasons is required to ensure that a similar agreement is achieved when the ponies have their winter coats. This is typically the time when the palpation of the animal is necessary to accurately determine body condition [41] (Henneke et al. 1983). However, in total, a total of 69 ponies were BCS assessed with good reliability, and 34 of the 69 trials were conducted during February and March when winter coats were not fully shed.

Our results showed that an initial trial of this prototype assessment achieved good reliability between assessors for many of the indicators. One limitation of the study was that our assessors only assessed the reliability of the welfare indicators in weather that did not include heavy rain or snow. This was due to time of year and safety considerations relating to visibility on the mountains. Further testing should be carried out under different weather conditions permissible within the mountain safety advice for Snowdonia National Park.

Another limitation of this study was that our trial was limited to one population of free-roaming Carneddau Mountain ponies and a relatively small sample size of individuals. Sample sizes have varied across similar studies; Viksten et al. 2017 tested their HWAP draft protocol for reliability on 37 horses from two Swedish riding schools using one assessor, while the Animal Welfare Indicators (AWIN) horse welfare assessment protocol was tested by researchers for reliability at 10 German horse farms, with a total of 435 horses being assessed by two assessors [35]. Nonetheless, this sample size reflected 16% of the total population, and this study’s aim of testing of AB and RB indicators for feasibility, reliability, and repeatability was therefore mostly achieved using this sample. This study therefore provides a useful baseline understanding for future testing and suitable evidence to be evaluated prior to the inclusion of these welfare indicators in welfare assessment templates for free-roaming horses. To assess the true potential of these indicators for free-roaming horses, further trialing will continue across different horse and pony populations. Additionally, testing will be carried out with practitioners (stock persons and grazing scheme managers), as the tool is ultimately intended for those working directly with the animals.

## 5. Conclusions

A range of welfare assessment protocols has been developed to determine the welfare of farm, companion, laboratory, and zoo animals. These objective appraisals and formal recordings of the welfare state of an animal have been found to contribute to welfare improvements in a myriad of species. As ours was a preliminary study, the focus was on the reliability, feasibility, and repeatability of indicators rather than an assessment of the ponies’ welfare per se. This study is therefore an initial step towards designing a much-needed tool for the assessment of free-roaming native ponies. Our preliminary assessment has demonstrated that many of the trialed welfare indicators were repeatable, with many featuring good reliability. It has also established that with minor modifications, animal-based indicators commonly used to assess equines in intensive conditions could be applied in free-roaming ponies. While the further refinement of the decision trees is required, the initial trial of the selected welfare indicators has enabled the identification of potential risks for welfare compromise in free-living Carneddau ponies. Results were presented to members of the Carneddau Pony Society to aid them in the necessary mitigations protocols to safeguard pony welfare.

## Figures and Tables

**Table 1:** List of the animal-based (AB) and resource-based (RB) welfare indicators chosen with details of possible outcomes for each assessed pony. The use of the HGS was only indicated as a second level assessment if a score of zero had been assigned for another indicator.

Score Category ^1^	0	1	2	3
Welfare Indicator				
Feeding/nutrition BCS Uses Henneke 9-point scale (AB)	Poor/very thin Fat/extremely fat	Thin/moderately thin	Moderate to fleshy	
Feeding/nutrition water availability (RB)		No water detected or detected but not accessible to the ponies (e.g., reservoir and fenced)	Pony has access to pooling water, puddle, or marsh area	Pony has access to fresh, free running stream, spring, or spring fed lake/pond
Environment human disturbance/ease of movement (RB)		Presence and proximity of the mountain bikers, unleashed dogs, walkers, runners, and campers that inhibit the pony’s movement	People are present, but they are not inhibiting the movement, pony can freely take another route	There are no people, dogs, or bikes present
Environment hazards fencing material, telephone wire, and manmade hazards/ease of movement (RB)		Hazards that inhibit the pony’s ability to move freely are present	Hazards are present, but the pony can avoid them and navigate around obstacles	No hazards are present
Environment resting comfort (RB)		Pony does not have a clean, dry quiet area to rest; it is muddy or very wet, and there is high human disturbance	Pony has a clean dry area, but there is high human disturbance	Pony has a clean dry area to lay down or rest, and there is no human disturbance
Environment thermal comfort (RB) access to shelter (AB) shivering/sweating		Pony does not have access to shelter or shade and/or is shivering or sweating	Pony does not have access to shelter or shade and is not shivering or sweating	Pony has access to shade and/or shelter and is not shivering or sweating
Health mobility (AB)	Immobile or severely impaired mobility; pony is unable to stay with herd	Walking with visible signs of abnormality, may showuneven weight-bearing in rest and/or walking, but the pony can stay with herd	No signs of abnormality	
Health skin condition mane/tail pruritis (itching) (AB)		Hair is missing and broken mane or tail	Normal mane and tail no evidence of hair loss	
Health skin/coat condition head, neck, body, and limbs (AB)		Coat is patchy or uneven (tufts of hair, e.g., winter coat visible out of season when other ponies shed). Skin may be visible (alopecia) in places.	Coat appears in good condition (not dull or dry , no hair loss is visible),	
Health ocular discharge (AB)	Discharge and partial or complete closure of the eye, with or without swelling.	Discharge with eye open includes mucus in eye and with visible discharge down cheek	Normal eye with no discharge	
Health nasal discharge (AB)		Nasal discharge present	No sign of nasal discharge	
Health fecal consistency (AB)	Water-like consistency (diarrhea)			
Health coughing (AB)		Pony has coughed at least once	Pony has not coughed	
Health wounds and swelling (AB)	Open wound, >7 cm involving deeper tissue (wound is not superficial), muscle, and/or tendon may be visible. Wound may be acute or is old but appears infected puss is visible/oozing. Bright green or yellow discharge or red skin adjacent to the wound	Wound is >7 cm, but it is healing (no puss, not bleeding, or acute) or pony has visible swelling, skin area > 7 cm with or without hair loss	Pony has no wounds or swollen areas of skin	
Behavior social contact (RB)		Solitary (no other ponies within visible range)	Other ponies present	
Behavior human approach test (AB)		Pony moves away when assessor is more than 9 m away	Pony moves away from assessor when assessor is less than 9 m away	Pony does not move away from assessor; assessor must stop at 3 m
Behavior HGS (2nd level if required) (AB)	HGS score: 8–12	HGS score: 4–7	HGS score: 0–3	

^1^ Score categories for welfare indicators. Zero or one represents a negative state of different degrees of severity, followed by two = neutral, and (sometimes) three = positive.

**Table 2:** Kappa weighted values and their 95% confidence intervals for all preliminary indicators tested for inter-observer reliability in a sample of 34 ponies. K is 1 when there was perfect agreement and 0 when there was no agreement better than chance. All κ_w_ values of 0.61 (good) and above are shown in bold.

Welfare Indicator	Percentage of Agreement	κ_w_ (95% CI)	Interpretation w/CI
BCS	97%	**0.78** (0.78–0.78	Good
Mobility	100%	**1.0** (1.0–1.0)	Very good

**Table 3:** κ_w_ estimates and their 95% confidence intervals for welfare indicators in the prototype assessment, which were tested for inter-assessor reliability between the primary investigator and each of the assessors. Assessor 1 (A1) (primary investigator) assessed all thirty-five ponies along with one of the other assessors (A2–A4). NT—data were not able to be tested due to an insufficient number of categories. All values of 0.61 (good) and above are shown in bold. κ_w_ is 1 when there was perfect agreement and 0 when there was no agreement better than chance. Interpretation is according to the work of Altman [54].

Welfare Indicator	Assessor Identity A1–A4	Percentage of Agreement	κ_w_ (95% CI)	Interpretation w/CI
BCS	1 and 2	100	**1.0** (1.0–1.0)	Very good
	1 and 3	83	0.57 (−0.12–1.0)	Poor–Moderate
	1 and 4	100	**1.0** (1.0–1.0)	Very good
Ease of movement (Hazards)	1 and 2	94	**0.89** (0.67–1.0)	Good–Very good
	1 and 3	100	-	NT
	1 and 4	100	-	NT
Comfort around resting	1 and 2	70	0.42 (0.07–0.76)	Poor–Moderate
	1 and 3	100	-	NT
	1 and 4	72	0.0	No agreement
Thermal environment and comfort	1 and 2	61	0.18 (0.25–0.61)	Poor
	1 and 3	100	-	NT
	1 and 4	81	0.0	No agreement
Social contact	1 and 2	100	**1.0** (1.0–1.0)	Very good
	1 and 3	100	**1.0** (1.0–1.0)	Very good
	1 and 4	90	**0.65** (0.4–1.0)	Poor–Good
Human approach test	1 and 2	80	**0.83** (0.61–1.0)	Very good
	1 and 3	81	**0.73** (0.32–1.0)	Fair–Good
	1 and 4	83	**0.85** (0.63–1.0)	Good–Very good

**Table 4:** Welfare scores for indicators with 100% agreement listed by assessor. These indicators were excluded from further analysis. Assessor 1 (A1) (primary investigator) assessed *n* = 35 ponies along with one of the other assessors; A2 (*n* = 18), A3 (*n* = 11), and A4 (*n* = 6).

Welfare Indicator	Score	A1	A2	A1	A3	A1	A4
Ocular discharge/swelling Score: 0–2	0 1 2	1 0 17	1 0 17	0 0 6	0 0 6	0 0 11	0 0 11
Mobility Score: 0–2	0 1 2	1 1 16	1 1 16	0 0 6	0 0 6	0 0 11	0 0 11
Skin/coat condition (head/body) Score: 1–2	1 2	2 16	2 16	0 6	0 6	0 11	0 11
Reproductive Status NA, A (lactating), or B (not lactating)	NA A B	9 2 7	9 2 7	3 1 2	3 1 2	5 2 4	5 2 4

**Table 5:** Kappa estimates and their 95% confidence intervals for welfare indicators in the prototype assessment (test/retest reliability). All kappa values of 0.61 (good) and above are shown in bold. Interpretation is according to the work of Altman [54].

Welfare Indicator	Percentage of Agreement	κ (95% CI)	Interpretation w/CI
BCS	100	**1.0** (1.0–1.0)	Very good
Reproductive status	100	**1.0** (1.0–1.0)	Very good
Resting comfort	90	0	No agreement
Ease of movement (people and dogs)	85	**0.67** (0.33–0.99)	Fair–Good
Ease of movement (hazards)	85	0.40 (0.11–0.87)	Poor–Moderate
Thermal environment	85	0.48 (0.10–0.85)	Poor–Moderate
Skin condition head, neck, body and limbs	100	**1.0** (1.0–1.0)	Very good
Wounds and swelling	95	**0.65** (0.25–1.0)	Fair–Good
Social contact	95	0–0	No agreement
Human approach	72	0.53 (0.19–0.87)	Poor–Moderate

## Data Availability

The data that support the findings of this study are available from the corresponding author, J.J.H., upon reasonable request.

## References

[1] Harvey A.M., Beausoleil N.J., Ramp D., Mellor D.J. (2020). A Ten-Stage Protocol for Assessing the Welfare of Individual Non-Captive Wild Animals: Free-Roaming Horses (Equus Ferus Caballus) as an Example. Animals.

[2] Knierim U., Winckler C. (2009). On-farm welfare assessment in cattle: Validity, reliability and feasibility issues and future perspectives with special regard to the Welfare Quality® approach. Anim. Welf..

[3] Munoz C., Campbell A., Hemsworth P., Doyle R. (2018). Animal-Based Indicators to Assess the Welfare of Extensively Managed Ewes. Animals.

[4] Ohl F., Putman R. (2018). The Biology and Management of Animal Welfare.

[5] Sterling P., Schulkin J. (2004). Principles of Allostasis: Optimal Design, Predictive Regulation, Pathophysiology, and Rational Therapeutics. Allostasis, Homeostasis, and the Costs of Physiological Adaptation.

[6] Whaytt H.R., Main D.C.J., Green L.E., Webster A.F.J. (2003). Animal-based methods for the assessment of welfare state of dairy cattle, pigs, and laying hens: Consensus of expert opinion. Anim. Welf..

[7] Battini M., Barbieri S., Vieira A., Stilwell G., Mattiello S. (2016). 2016 Results of testing the prototype of the AWIN welfare assessment protocol for dairy goats in 30 intensive farms in Northern Italy. Ital. J. Anim. Sci..

[8] Welfare Quality (2009). Assessment Protocol for Cattle.

[9] Barnard S., Pedernera C., Candeloro L., Ferri N., Velarde A., Dalla Villa P. (2016). 2016 Development of a new welfare assessment protocol for practical application in long-term dog shelters. Vet. Rec..

[10] Hawkins P., Morton D.B., Burman O., Dennison N., Honess P., Jennings M., Lane S., Middleton V., Roughan J.V., Wells S. (2011). A guide to defining and implementing protocols for the welfare assessment of laboratory animals: Eleventh report of the BVAAWF/FRAME/RSPCA/UFAW Joint Working Group on Refinement. Lab. Anim..

[11] Harley J., Clark F.E. (2019). BIAZA Animal Welfare Toolkit.

[12] Yon L., Williams E., Harvey N.D., Asher L. (2019). Development of a behavioral welfare assessment tool for routine use with captive elephants. PLoS ONE.

[13] Wolfensohn S., Shotton J., Bowley H., Davies S., Thompson S., Justice W.S. (2018). Assessment of welfare in zoo animals: Towards optimum quality of life. Animals.

[14] Kagan R., Carter S., Allard S. (2015). A Universal Animal Welfare Framework for Zoos. J. Appl. Anim. Welf. Sci..

[15] Kaurivi Y., Laven R., Hickson R., Stafford K., Parkinson T. (2019). Identification of suitable animal welfare assessment indicators for extensive beef systems in New Zealand. Agriculture.

[16] Turner S.P., Dwyer C.M. (2007). Welfare assessment in extensive animal production systems: Challenges and opportunities. Anim. Welf..

[17] Hobbs R.J., Hinds L.A. (2018). Could current fertility control methods be effective for landscape-scale management of populations of wild horses (*Equus caballus*) in Australia?. Wildl. Res..

[18] (2020). BLM. www.blm.gov/plrograms/wild-horse-and-burro/about-the-program/program-data.

[19] Dalla Costa E., Murray L., Dai F., Canali E., Minero M. (2014). Equine on-farm welfare assessment: A review of animal-based indicators. Anim. Welf..

[20] Horseman S.V., Buller H., Mullan S., Whay H.R. (2016). Current welfare problems facing horses in Great Britain as identified by equine stakeholders. PLoS ONE.

[21] Spratling B. (2012). Bureau of Land Management Wild Horse and Burro Program: Welfare on the Range. Proc. Am. Assoc. Equine Pract..

[22] Pritchard J.C., Lindberg A.C., Main D.C., Whay H.R. (2005). Assessment of the welfare of working horses, mules and donkeys, using health and behaviour parameters. Prev. Vet. Med..

[23] Livestock Research Welfare Monitoring System: Assessment Protocol for Horses (Rapport/Wageningen UR Livestock Research; No. 569). 2012 Wageningen UR Livestock Research. https://edepot.wur.nl/.

[24] Dalla Costa E., Dai F., Lebelt D., Scholz P., Barbieri S., Canali E., Minero M. (2016). Welfare assessment of horses: The AWIN approach. Anim. Welf..

[25] Viksten S., Visser E., Nyman S., Blokhuis H. (2017). Developing a horse welfare assessment protocol. Anim. Welf..

[26] Sommerville R., Brown A.F., Upjohn M. (2018). A standardised equine-based welfare assessment tool used for six years in low and middle income countries. PLoS ONE.

[27] Fraser M.D., Stanley C.R., Hegarty M.J. (2019). Recognising the potential role of native ponies in conservation management. Biol. Conserv..

[28] Stanley C.R., Mettke-Hofmann C., Hager R., Shultz S. (2018). Social stability in semiferal ponies: Networks show interannual stability alongside seasonal flexibility. Anim. Behav..

[29] Winton C.L., Hegarty M.J., McMahon R., Slavov G.T., McEwan N.R., Davies-Morel M.C., Morgan C.M., Powell W., Nash D.M. (2013). Genetic diversity and phylogenetic analysis of native mountain ponies of Britain and Ireland reveals a novel rare population. Ecol. Evol..

[30] Stanley C.R. (2018). Carneddau Ponies—Advice on Potential for Seeking Rare Breed Status.

[31] Dalla Costa E., Minero M., Lebelt D., Stucke D., Canali E. (2014). Development of the Horse Grimace Scale (HGS) as a Pain Assessment Tool in Horses Undergoing Routine Castration. PLoS ONE.

[32] Mullan S., Szmaragd C., Hotchkiss J., Whay H.R. (2014). The welfare of long-line tethered and free-ranging horses kept on public grazing land in South Wales. Anim. Welf..

[33] AWIN (2015). AWIN Welfare Assessment Protocol for Horses.

[34] Viksten S., Visser E.K., Blokhuis H. (2016). A comparative study of the application of two horse welfare assessment protocols. Acta Agric. Scand. Anim. Sci..

[35] Czycholl I., Klingbeil P., Krieter J. (2019). Interrater Reliability of the Animal Welfare Indicators Welfare Assessment Protocol for Horses. J. Equine Vet. Sci..

[36] Lesimple C. (2020). Indicators of horse welfare: State-of-the-art. Animals.

[37] Reed S.M., Bayly W.M., Sellon D.C. (2018). Equine Internal Medicine.

[38] AAEP Lameness Exams: Evaluating the Lame Horse. https://aaep.org/horsehealth/lameness-exams-evaluating-lame-horse.

[39] Agriculture and Horticulture Development Board Mobility Scoring for Dairy Cows. https://ahdb.org.uk/knowledge-library/mobility-scoring-for-dairy-cows.

[40] Carroll C.L., Huntington P.J. (1988). 1988 Body condition scoring and weight estimation of horses. Equine Vet. J..

[41] Henneke D.R., Potter G.D., Kreider J.L., Yeates B.F. (1983). Relationship between condition score, physical indicatorments and body fat percentage in mares. Equine Vet. J..

[42] Debeffe L., Mcloughlin P.D., Medill S.A., Stewart K., Andres D., Shury T., Wagner B., Jenkins E., Gilleard J.S., Poissant J. (2016). Negative covariance between parasite load and body condition in a population of feral horses. J. Parasitol..

[43] Cain J.L., Jarisch K., Macaluso K.R., Luedtke B.E. (2018). Correlation between fecal egg count, presence of Strongylus vulgaris, and body score of feral horses on Fort Pulk, Louisiana. Vet. Parasitol. Reg. Stud. Rep..

[44] Rudman R., Keiper R.R. (1991). The body condition of feral ponies on Assateague Island. Equine Vet. J..

[45] Merskey H., Bogduk N., ISAP (1994). Part III: Pain Terms, A Current List with Definitions and Notes on Usage Classification of Chronic Pain.

[46] Hellebrekers L.J. (2000). Pathophysiology of Pain in Animals and Its Consequence for Analgesic Therapy in Animal Pain: A Practice-Oriented Approach to an Effective Pain Control in Animals.

[47] McLennan K.M., Rebelo C.J., Corke M.J., Holmes M.A., Leach M.C., Constantino-Casas F. (2016). Development of a facial expression scale using footrot and mastitis as models of pain in sheep. App. Anim. Behav. Sci..

[48] Keating S.C.J., Thomas A.A., Flecknell P.A., Leach M.C. (2012). Evaluation of EMLA cream for preventing pain during tattooing of rabbits: Changes in physiological, behavioral and facial expression responses. PLoS ONE.

[49] Sotocinal S.G., Sorge R.E., Zaloum A., Tuttle A.H., Martin L.J., Wieskopf J.S. (2011). The Rat Grimace Scale: A partially automated method for quantifying pain in the laboratory rat via facial expressions. Mol. Pain.

[50] R Core Team (2020). R: A Language and Environment for Statistical Computing.

[51] (2020). MedCalc Statistical Software Version 19.2.3.

[52] Cohen J. (1960). A coefficient of agreement for nominal scales. Educ. Psychol. Meas..

[53] Cohen J. (1968). Weighted kappa: Nominal scale agreement provision for scaled disagreement or partial credit. Psychol. Bull..

[54] Altman D.G. (1991). Practical Statistics for Medical Research.

[55] McHugh M.L. (2012). Interrater reliability: The kappa statistic. Biochem. Med..

[56] Revelle W. (2020). Psych: Procedures for Personality and Psychological Research.

[57] Lewis J.S., Spaulding S., Swanson H., Keeley W., Gramza A.R., VandeWoude S., Crooks K.R. (2021). Human activity influences wildlife populations and activity patterns: Implications for spatial and temporal refuges. Ecosphere.

[58] Brooks D.E., Matthews A.G. (1999). Equine ophthalmology. Vet. Ophthalmol..

[59] HadUKP Monthly Northwest England & Wales Precipitation (mm). https://www.metoffice.gov.uk/hadobs/hadukp/data/monthly/HadNWEP_monthly_qc.txt.

[60] Alexander L.V., Jones P.D. (2001). Updated precipitation series for the UK and discussion of recent extremes. Atmos. Sci. Lett..

[61] Stemler S.E. (2004). A Comparison of Consensus, Consistency, and Measurement Approaches to Estimating Interrater Reliability. Pract. Assess. Res. Eval..

[62] Viera A.J., Garrett J.M. (2005). Understanding interobserver agreement: The kappa statistic. Fam. Med..

[63] Gwet K.L. (2014). Handbook of Inter-Rater Reliability: The Definitive Guide to Measuring the Extent of Agreement among Assessors.

[64] Kristensen E., Dueholm L., Vink D., Andersen J.E., Jakobsen E.B., Illum-Nielsen S., Petersen F.A., Enevoldsen C. (2006). Within-and across-person uniformity of body condition scoring in Danish Holstein cattle. Int. J. Dairy Sci..

[65] Stanley C.R. (2015). Influences of Kinship, Social Bonds and Genetics on Animal Social Structure.

